# Steroid alterations in Cushing’s disease persist after remission: ACTH-driven steroid changes dissociate from mood-regulatory neurosteroids

**DOI:** 10.3389/fendo.2026.1829888

**Published:** 2026-07-02

**Authors:** Sebnem Burhan, Ebubekir Akpinar, Sevim Eyupoglu, Ayhan Bingol, Merve Korkmaz Yilmaz, Aslihan Pekmezci, Ebru Turgal, Huseyin Sehit Burhan, Mutlu Niyazoglu, Esra Suheda Hatipoglu

**Affiliations:** 1 Division of Endocrinology, Department of Internal Medicine, University of Health Sciences, Basaksehir Cam and Sakura City Hospital, Istanbul, Türkiye; 2 Department of Neurosurgery, University of Health Sciences, Basaksehir Cam and Sakura City Hospital, Istanbul, Türkiye; 3 University of Health Sciences, Pituitary Disorders Application and Research Center (HATMER), Istanbul, Türkiye; 4 Department of Psychology, Atlas University, Istanbul, Türkiye; 5 Department of Psychology, Davranis Degisim Akademia, Istanbul, Türkiye; 6 Strathclyde Institute of Pharmacy and Biomedical Sciences, Glasgow, United Kingdom; 7 Department of Psychiatry, University of Health Sciences, Basaksehir Cam and Sakura City Hospital, Istanbul, Türkiye

**Keywords:** ACTH, Cushing’s disease, dehydroepiandrosterone, depression, mass spectrometry, neurosteroids

## Abstract

**Objective:**

Cushing’s disease (CD) is associated with high rates of depression and anxiety that often persist despite biochemical remission, yet the underlying mechanisms remain unclear. This study aims to characterize circulating neurosteroid (NS) profiles in CD and examine their associations with psychological symptoms.

**Design and methods:**

This is a cross-sectional study of 37 patients with CD (22 active, 15 remission) and 21 nonfunctioning pituitary adenoma (NFA) controls. NS levels were quantified by mass spectrometry; psychological symptoms were assessed using the 21-item Depression Anxiety Stress Scale (DASS-21). Discriminatory NS were identified by partial least squares discriminant analysis (PLS-DA), with a variable importance in projection (VIP) score > 1.0 used as the selection threshold.

**Results:**

Patients with CD exhibited higher depression (14 vs. 7.5, *p* = 0.03) and stress scores (17 vs. 8.5, *p* = 0.03) than NFA. PLS-DA identified five discriminatory NS: 4-androstenedione (VIP = 2.0), 11-deoxycorticosterone (DOC) (VIP = 1.8), 7-OH-pregnenolone (VIP = 1.8), corticosterone (VIP = 1.7), and androsterone (VIP = 1.1); the first four were elevated, and androsterone decreased in CD. Despite biochemical remission, most NS alterations persisted, with 7-OH-pregnenolone paradoxically increasing further (121 vs. 22.5 ng/mL, *p* = 0.008). Mood symptoms did not correlate with cortisol, adrenocorticotropic hormone (ACTH), or urinary free cortisol (UFC). However, dehydroepiandrosterone (DHEA) correlated positively with depression (*r* = 0.4, *p* = 0.02) and stress (*r* = 0.4, *p* = 0.02), whereas allopregnanolone correlated negatively with depression (*r* = −0.4, *p* = 0.03) and stress (*r* = −0.4, *p* = 0.04). Among discriminatory NS, only 7-OH-pregnenolone correlated with stress (*r* = 0.3, *p* = 0.04). These NS–mood associations were absent in NFA.

**Conclusions:**

CD is characterized by a distinct and persistent steroid signature that persists beyond biochemical remission, reflecting hypercortisolism-driven epigenetic remodeling of steroidogenic pathways. The dissociation between ACTH-driven steroid alterations and mood-associated NS suggests that cortisol reduction alone may inadequately address psychiatric symptoms, warranting investigation of NS-targeted therapies.

## Introduction

Cushing’s disease (CD) is strongly associated with a high prevalence of psychiatric disorders, with major depression affecting 50%–81% of patients with active disease and anxiety disorders impacting up to 66% (1). These symptoms substantially impair quality of life and often persist in more than 20% of patients even after successful treatment and biochemical remission (2, 3). Consequently, many patients experience prolonged psychological distress that requires ongoing psychiatric support and significantly impacts long-term outcomes. However, the neurobiological mechanisms underlying persistent mood disorders following CD remission remain poorly understood, and effective treatments beyond conventional antidepressants are limited. Identifying novel mechanistic pathways and potential therapeutic targets for CD-associated psychiatric morbidity therefore represents an important unmet clinical need.

Neurosteroids (NS) are cholesterol-derived neuroactive molecules synthesized *de novo* in the central nervous system or imported from peripheral circulation that potently modulate neuronal excitability, synaptic plasticity, and affective behavior (4). These molecules exert rapid effects primarily through non-genomic mechanisms, acting as allosteric modulators of neurotransmitter receptors and ion channels, altering neural activity within seconds to minutes (4–6). NS levels fluctuate across physiological states, including stress, menstrual cycle phases, pregnancy, and aging, and are dysregulated in various neuropsychiatric conditions, including major depression, anxiety disorders, and post-traumatic stress disorder (6). Although the term “NS” originally referred to steroids synthesized *de novo* in the central nervous system, we use “NS” broadly to encompass peripherally measured steroids with neuroactive properties. Throughout this manuscript, we use “steroid” to refer to adrenocorticotropic hormone (ACTH)-driven adrenal synthesis and metabolic profiles, and “NS” to emphasize neuroactive or mood-regulatory properties, recognizing that these represent functional rather than structural distinctions. Critically, circulating NS can cross the blood–brain barrier to modulate central nervous system function, rendering peripheral measurements clinically relevant for assessing NS tone and mood regulation (7). The steroids measured in our study likely originate predominantly from ACTH-driven adrenal synthesis.

Allopregnanolone and pregnanolone are NS that function as positive allosteric modulators of GABA-A receptors, enhancing inhibitory neurotransmission and playing critical roles in stress response regulation through the hypothalamic–pituitary–adrenal (HPA) axis (7–9). Reduced concentrations of allopregnanolone in plasma, serum, and cerebrospinal fluid have been consistently reported in patients with major depression, often correlating inversely with symptom severity (8, 10). Another NS, dehydroepiandrosterone (DHEA), exhibits neuroprotective, anxiolytic, and antidepressant properties (11). The therapeutic potential of NS has gained substantial clinical traction, with the U.S. Food and Drug Administration (FDA) and European Medicines Agency (EMA) approving the GABA-A modulator brexanolone (synthetic allopregnanolone) for moderate-to-severe postpartum depression (12). Additional NS-based therapies are under investigation for major depression, bipolar disorder, and other psychiatric conditions (13, 14).

Although direct evidence linking NS alterations to CD is currently lacking, several lines of indirect evidence suggest potential mechanistic connections. Glucocorticoids in CD act on mineralocorticoid and glucocorticoid receptors in limbic brain regions enriched in GABAergic and glutamatergic signaling systems, the primary targets of NS modulation (15–17). Furthermore, experimental models of chronic hypercortisolism demonstrate neuronal atrophy in hippocampal regions, altered dendritic morphology, and impaired glutamate neurotransmission, all of which may interact with disrupted GABAergic NS tone (18). Additionally, cortisol and NS share common biosynthetic pathways from cholesterol, suggesting that chronic hypercortisolism may alter NS synthesis, thereby contributing to mood dysregulation.

Despite these mechanistic connections and the established role of NS in mood disorders, no studies have directly measured circulating NS levels in patients with CD or examined their relationship with psychiatric symptoms. We therefore conducted this study to (i) characterize circulating concentrations of key NS in patients with CD and a control group of patients with nonfunctioning pituitary adenoma (NFA); (ii) examine associations between NS levels and validated measures of depression, anxiety, and perceived stress in patients with CD.

## Materials and methods

### Study population and procedures

This cross-sectional study included 37 patients with CD (22 active, 15 remission) and 21 age- and sex-matched NFA controls from a tertiary endocrinology center (May 2023–August 2024). NFA controls were selected to account for potential confounding effects of pituitary mass lesions and surgical candidacy.

CD diagnosis was confirmed by laboratory evidence of hypercortisolism [elevated 24-h urinary free cortisol (UFC), late-night salivary cortisol, and/or post-dexamethasone serum cortisol >1.8 μg/dL], elevated ACTH, pituitary adenoma on magnetic resonance imaging, and exclusion of ectopic ACTH sources. Remission was defined as normalization of UFC and post-dexamethasone cortisol <1.8 μg/dL. Exclusion criteria included cerebrovascular disease, psychiatric disorders, psychotropic medication use, and exogenous corticosteroid therapy (except physiological replacement for secondary adrenal insufficiency).

The study was approved by the institutional ethics committee and conducted in accordance with the Declaration of Helsinki.

### Laboratory evaluation

Fasting venous blood samples (08:00–10:00) were collected and stored at −80 °C until analysis. NS concentrations were quantified using liquid chromatography–tandem mass spectrometry (LC-MS/MS; Agilent 1290 HPLC coupled to a 6470 triple quadrupole mass spectrometer). The core panel of 16 NS was measured using the validated Jasem Steroid Hormones kit (JSM-CL-6500), which quantifies aldosterone, androstenedione, androsterone, corticosterone, cortisol, DHEA, dehydroepiandrosterone sulfate (DHEAS), dihydrotestosterone, estradiol (E2), 11-deoxycorticosterone (DOC), 11-deoxycortisol, 17α-OH-pregnenolone, 17-OH-progesterone, pregnenolone, progesterone, and testosterone. Two additional NS, 7-OH-pregnenolone and allopregnanolone, were quantified using a validated commercial add-on module (Jasem) on the same LC-MS/MS platform, employing dedicated internal standards and calibrators specific to each analyte. In total, a panel of 18 NS was measured.

### Psychological assessment

Depression, anxiety, and stress were assessed using the validated 21-item Depression Anxiety Stress Scale (DASS-21) (19, 20), with higher scores indicating greater symptom severity.

### Statistical analysis

Analyses were performed using SPSS 25.0 and MetaboAnalyst 6.0. Normality was assessed via the Shapiro–Wilk test. Group comparisons used the *t*-test or the Mann–Whitney *U* test for continuous variables and the chi-square test for categorical variables. Spearman correlation assessed variable relationships. Partial least squares discriminant analysis (PLS-DA) identified discriminatory NS after auto-scaling and imputation of below-detection values with half the minimum detectable concentration. Variables with variable importance in projection (VIP) scores > 1.0 were considered significant discriminators. Statistical significance was set at *p* < 0.05.

## Results

The study included 37 patients with CD and 21 controls with NFA. The groups were comparable in terms of age, sex distribution, and education level (*p* = 0.5, *p* = 0.9, and *p* = 0.3, respectively). **Table 1** displays a comparison of the groups’ demographic and laboratory information. The additional clinical characteristics of the cases with CD are presented in **Table 2**.

**Table 1:** Demographic characteristics of the groups.

Characteristic	CD (*n* = 37)	NFA (*n* = 21)	p-value
Age	48.2 ± 11.7	45.8 ± 15.1	0.5
Gender			0.9
*Male*	19% (*n* = 7)	19% (*n* = 4)	
*Female*	81% (*n* = 30)	81% (*n* = 17)	
Education			0.3
Illiterate	5.4% (*n* = 2)	**-**	
Primary school	59.5% (*n* = 22)	71.4% (*n* = 15)	
High school	24.3% (*n* = 9)	14.3% (*n* = 3)	
*College*	10.8% (*n* = 4)	14.3% (n = 3)	
Hypopituitarism			0.05
Adrenocortical	13.5% (*n* = 5)	–	
Adrenocortical and gonadal	2.7% (*n* = 1)	–	
Comorbidities			0.6
*Diabetes*	51% (*n* = 19)	19% (*n* = 4)	
*Hypertension*	22% (*n* = 22)	23% (*n* = 5)	
ACTH, ng/L	45.5 [27.7–71.4]	22.3 [16.8–26.9]	**0.004**
1 mg DST, µg/dL	2.9 [0.81–7.40]	0.8 [0.50–1.08]	**<0.001**
UFC, µg/24 h	34.0 [13.0–124]	–	

Data are presented as mean ± SD or number (%), median [IQR], unless otherwise indicated.

ACTH, adrenocorticotropic hormone; CD, Cushing’s disease; DST, dexamethasone suppression test; NFA, nonfunctioning pituitary adenoma; UFC, urinary free cortisol. Bold values indicate statistically significant differences (p<0.05).

**Table 2:** Clinical characteristics of patients with Cushing’s disease.

Disease duration (months)	30.3 ± 2.5
Treatment history	
*Newly diagnosed or pretreatment*	*n* = 16 (43.2%)
*Surgery*	*n* = 21 (56.7%)
*Gamma-knife*	*n* = 4 (10.8%)
*Ketoconazole*	*n* = 1 (2.7%)
*Metyrapone*	*n* = 2 (5.4%)
*Pasireotide*	*n* = 3 (8.1%)
Remission	*n* = 15 (40.5%)

Data are presented as mean ± SD or number (%), unless otherwise indicated.

### NS levels

The NS levels in both groups are presented in **Table 3**. The median levels of cortisol, corticosterone, dihydrotestosterone, 4-androstenedione, E2, and 7-OH-pregnenolone were significantly higher in the CD group (*p* = 0.01, *p* = 0.001, *p* = 0.01, *p* < 0.001, *p* < 0.001, and *p* = 0.002, respectively). Conversely, androsterone levels were significantly lower in the CD group (*p* = 0.01). The levels of other NS showed no variation between the groups (*p* > 0.05 for all comparisons). Additionally, NS levels showed no sex-related differences (*p* > 0.05 for all) except testosterone, which was higher in men within the CD group (*p* < 0.05).

**Table 3:** Comparison of serum NS levels between the groups.

Neurosteroids	CD (n = 37)	NFA (n = 21)	p-value
Pregnenolone	3.14 [1.8–8.3]	6.23 [4.87–6.93]	0.3
Progesterone	0.069 [0.039–0.16]	0.081 [0.039–0.186]	0.9
17α-OH-pregnenolone	0.961 [0.624–1.98]	0.569 [0.397–0.865]	0.1
7-OH-pregnenolone	62.47 [1.77–121]	1.69 [1.21–2.1]	**0.002**
17-OH-progesterone	0.228 [0.167–0.527]	0.246 [0.166–0.422]	0.8
DOC	0.052 [0.035–0.076]	0.038 [0.029–0.052]	0.1
Corticosterone	1.23 [0.88–2.56]	0.26 [0.106–1.25]	**0.001**
Aldosterone	0.05 [0.021–0.082]	0.05 [0.03–0.087]	0.9
11-Deoxycortisol	0.418 [0.187–0.618]	0.506 [0.301–1.72]	0.1
Cortisol	80.3 [50.5–105]	55.1 [38.8–72]	0.01
DHEA	1.27 [0.611–2.76]	1.03 [0.475–2.87]	0.6
DHEAS	556 [323–769]	533 [185–821]	0.7
4-Androstenedione	0.64 [0.39–0.96]	0.02 [0.01–0.41]	**<0.001**
Testosterone	0.16 [0.095–0.338]	0.214 [0.115–0.41]	0.3
Dihydrotestosterone	0.252 [0.086–0.341]	0.15 [0.051–0.161]	0.01
Androsterone	0.762 [0.449–0.968]	1.07 [0.681–1.90]	0.01
E2	0.703 [0.294–0.923]	0.14 [0.069–0.20]	<0.001
Allopregnanolone	1.01 [0.441–2.08]	0.55 [0.329–0.807]	0.2

Data are presented as median [IQR].

Levels: ng/mL.

CD, Cushing’s disease; DHEAS, dehydroepiandrosterone sulfate; DHEA, dehydroepiandrosterone; NFA, nonfunctioning pituitary adenomas; DOC, 11-deoxycorticosterone; E2, estradiol. Bold values indicate statistically significant differences (p<0.05).

**Figure 1:**
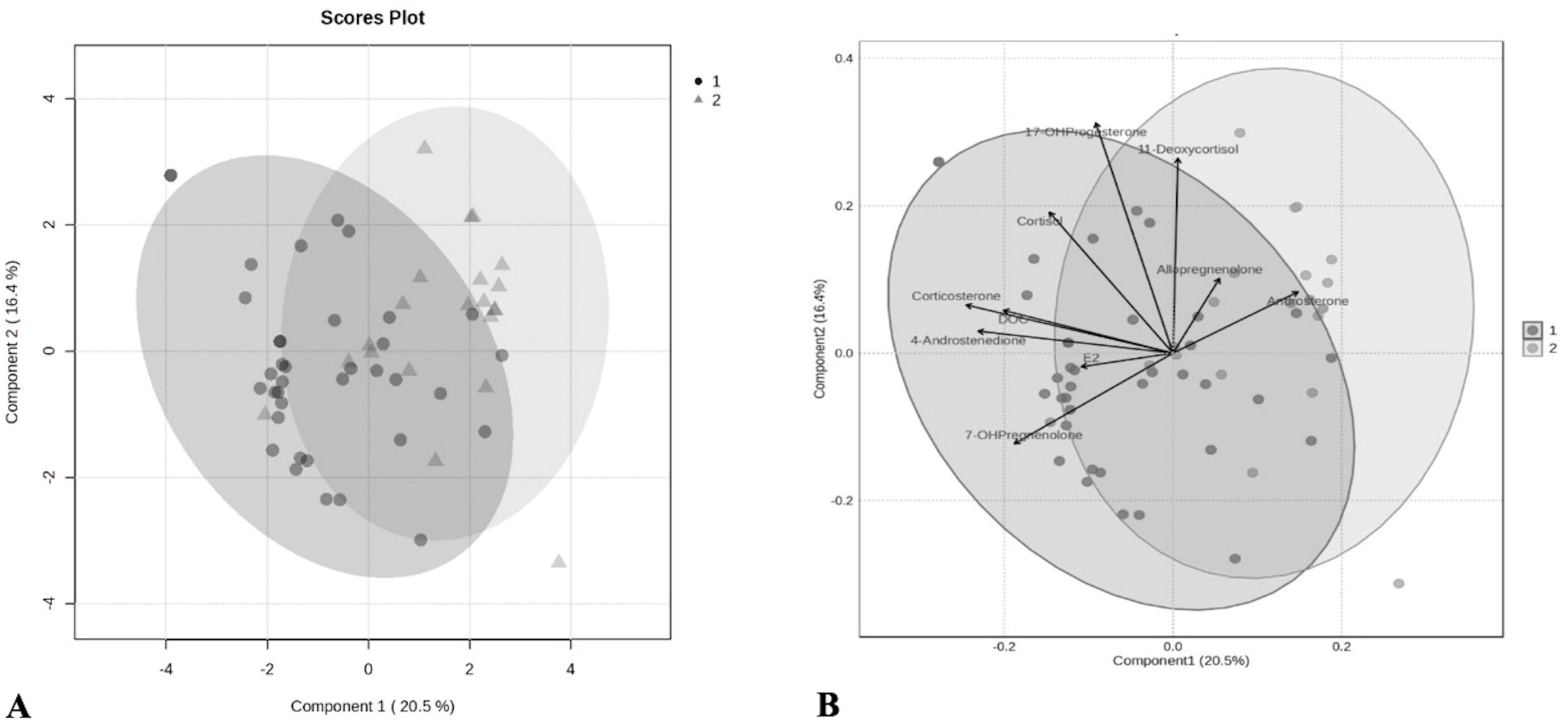
Multivariate analysis of serum NS. **(A)** PLS-DA score plot showing the separation of groups based on their steroidogenic profiles, Component 1: CD, Component 2: NFA. **(B)** Loading plot highlighting the contribution of individual NS to the components, Component 1: CD, Component 2: NFA, DOC: 11-deoxycorticosterone, E2: estradiol.

PLS-DA was performed to identify which NS are most prominent in each group. The PLS-DA score plot demonstrated separation between patients with CD and the NFA group, with Component 1 and Component 2 accounting for 20.5% and 16.4% of the total variance, respectively (**Figure 1**). VIP scores identified seven NS with VIP values > 1. The highest scores were observed for 4-androstenedione (VIP = 2), DOC (VIP = 1.8), 7-OH-pregnenolone (VIP = 1.8), corticosterone (VIP = 1.7), and androsterone (VIP = 1.1). Except for androsterone, the four other metabolites were elevated in the CD group.

Correlation analyses were performed between the NS with VIP scores > 1 and biochemical markers of disease activity (UFC and ACTH). UFC levels showed positive correlations with 4-androstenedione (*r* = 0.4, *p* = 0.03). ACTH levels correlated positively with 4-androstenedione (*r* = 0.5, *p* = 0.004) and negatively with 7-OH-pregnenolone (*r* = −0.5, *p* = 0.002). Other NS showed no significant correlation with UFC or ACTH levels (*p* > 0.05 for all).

To evaluate the effect of remission on NS levels, patients with CD were stratified by remission status (*n* = 22 active disease, *n* = 15 in remission). As expected, patients in remission showed significantly lower levels of ACTH (median: 21.2 vs. 64.5 pg/mL, *p* = 0.001), UFC (median: 12.6 vs. 86.5 μg/24h, *p* = 0.001), 1-mg dexamethasone suppression test (DST) (median: 0.8 vs. 6.5 μg/dL, *p* = 0.001), and cortisol (median: 50.5 vs. 88.3 ng/mL, *p* = 0.01). Among the NS with VIP scores > 1, only 7-OH-pregnenolone showed a significant difference between groups (median: 121.0 vs. 22.5 ng/mL, *p* = 0.008), with higher levels observed in the remission group. The remaining NS, including 4-androstenedione, corticosterone, DOC, and androsterone, showed no significant changes with remission status (*p* > 0.05 for all).

### Psychological assessment

The median DASS-21 depression scores were significantly higher in the CD group than in the NFA group [14 (IQR: 6–23) vs. 7.5 (IQR: 2–12.3), *p* = 0.03]. Similarly, median stress scores were higher in the CD group [17 (IQR: 8.5–25) vs. 8.5 (IQR: 2.5–16); *p* = 0.03]. There was no statistically significant difference in anxiety scores between the groups [8 (IQR: 2.5–19) vs. 7 (IQR: 2–13), *p* = 0.2].

Among patients with CD, depression, anxiety, and stress scores did not significantly differ when stratified by remission status, sex, history of radiotherapy, presence of hypopituitarism, comorbid conditions, or use of medical therapy. Moreover, none of the DASS-21 subscale scores correlated with basal cortisol, ACTH, or UFC levels, or with disease duration (*p* > 0.05 for all comparisons).

### Correlations of NS levels with the psychological status

**Figure 2:**
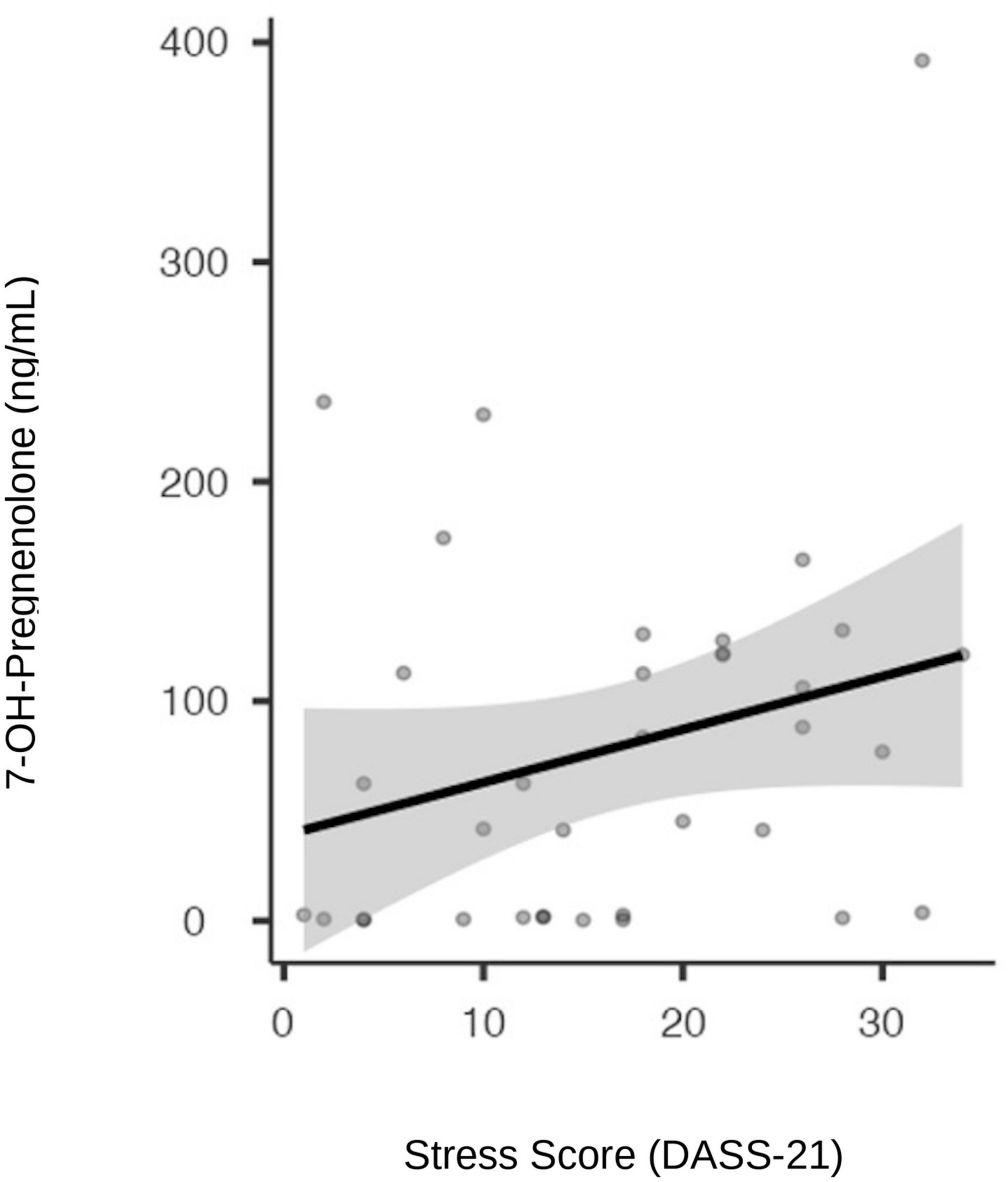
Correlation between serum 7-OH-pregnenolone levels and the scores on stress in cases with CD. Stress scores are shown on the *x*-axis and 7-OH-pregnenolone levels are shown on the *y*-axis. (*r* = 0.3, *p* = 0.04).

The relationship between NS identified by PLS-DA (VIP > 1) and psychological symptoms was evaluated in patients with CD. Among all NS, only 7-OH-pregnenolone showed a significant correlation with DASS-21 stress scores (*r* = 0.3, *p* = 0.04) (**Figure 2**). Regarding the NS characterizing the CD profile, no significant correlations were observed between corticosterone, 4-androstenedione, 7-OH-pregnenolone, or androsterone and depression/anxiety scores. Notably, no significant relationship was observed between cortisol levels and depression, anxiety, or stress scores (*p* > 0.05 for all).

**Figure 3:**
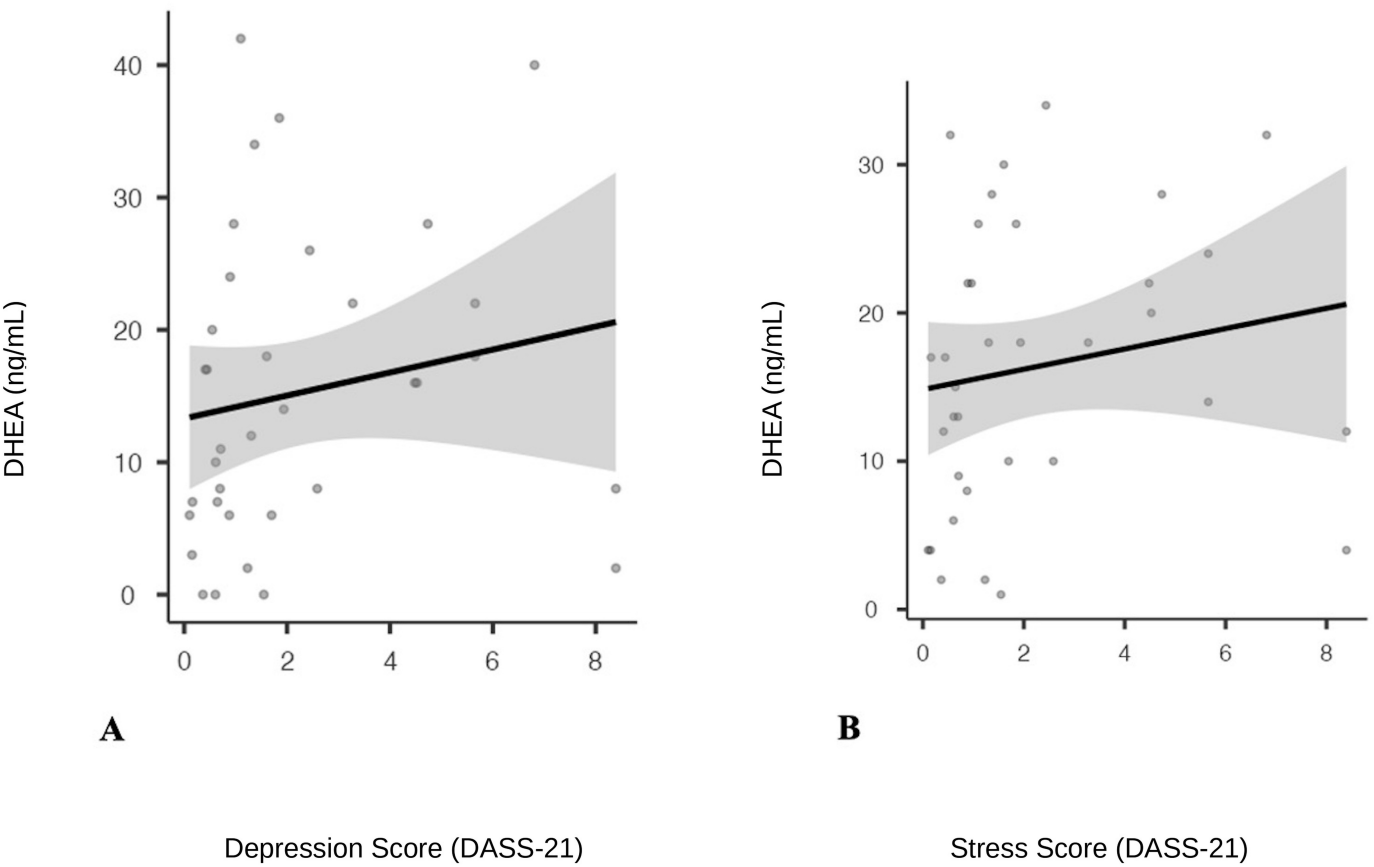
Correlation of the scores on depression and stress with DHEA levels in CD. Depression/stress scores are shown on the *x*-axis and DHEA levels are shown on the *y*-axis. **(A)** Relationship between depression scores and DHEA levels (*r* = 0.4, *p* = 0.02). **(B)** Relationship between stress scores and DHEA levels (*r* = 0.4, *p* = 0.02).

**Figure 4:**
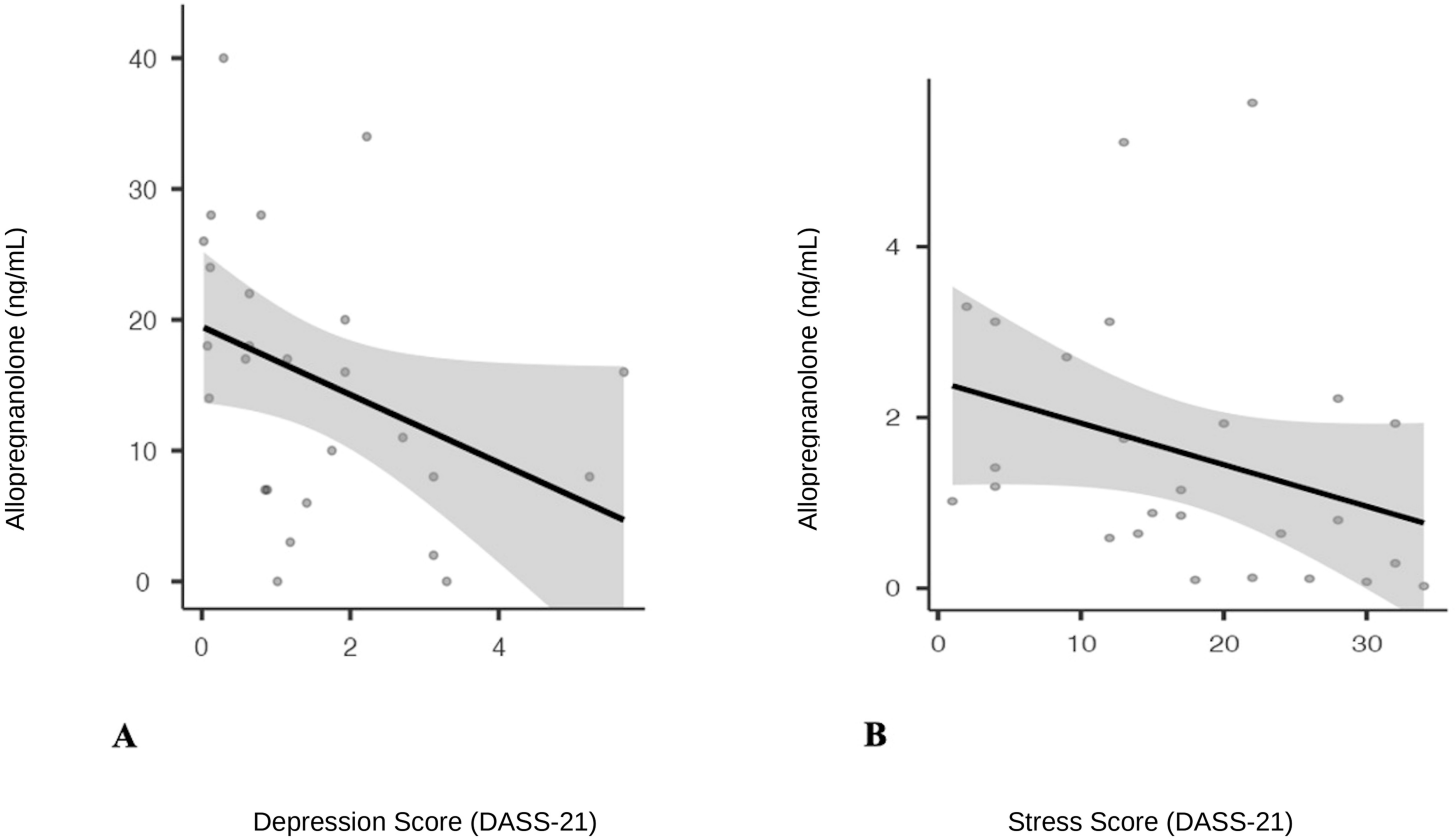
Correlation of the scores on depression and stress with allopregnanolone levels in CD. Depression/stress scores are shown on the *x*-axis and allopregnanolone levels are shown on the *y*-axis. **(A)** Relationship between depression scores and allopregnanolone levels (*r* = −0.4, *p* = 0.03). **(B)** Relationship between stress scores and allopregnanolone levels (*r* = −0.4, *p* = 0.04).

Additionally, the relationship between other NS and psychological symptoms was examined. DHEA levels showed positive correlations with both depression (*r* = 0.4, *p* = 0.02) and stress scores (*r* = 0.4, *p* = 0.02) (**Figure 3**). Conversely, allopregnanolone levels were negatively correlated with depression (*r* = −0.4, *p* = 0.03) and stress scores (*r* = −0.4, *p* = 0.04) (**Figure 4**). No significant correlations were observed between anxiety scores and any NS levels (*p* > 0.05 for all).

Of note, in cases with NFA, no statistically significant associations were found between NS and depression, stress, or anxiety (*p* > 0.05 for all, data not shown here).

## Discussion

In this study, patients with CD demonstrated significant alterations in steroid profiles accompanied by higher depression and stress scores, compared to patients with NFA. Using PLS-DA, 4-androstenedione, 7-OH-pregnenolone, and corticosterone were found to be increased in CD, while androsterone levels were reduced. Following remission, cortisol levels decreased as expected; however, other steroids, except 7-OH-pregnenolone, remained elevated in cases with CD. Interestingly, 7-OH-pregnenolone levels increased further during remission. Disease-related characteristics and markers of disease burden were not associated with mood symptoms in CD. Among NS, 7-OH-pregnenolone correlated positively with stress, DHEA correlated positively with both depression and stress, whereas allopregnanolone correlated negatively with depression and stress in patients with CD. In contrast, no NS–mood associations were observed in the NFA group.

To our knowledge, this is the first study to characterize the NS profile in ACTH-dependent CD comprehensively. NS are endogenous neuroactive molecules that modulate brain function, influencing neuronal excitability, neuroplasticity, and behavior (21). These compounds play a critical role in regulating the HPA axis. Allopregnanolone, pregnenolone, and DHEA are rapidly synthesized in response to acute stress and provide inhibitory feedback to the HPA axis, facilitating the restoration of homeostasis (7, 22, 23). Conversely, chronic stress or sustained hypercortisolism suppresses NS synthesis and disrupts HPA axis regulation (7, 22).

Masjkur et al. demonstrated that distinct steroid profiles can differentiate subclinical from overt adrenal Cushing’s syndrome (24). In adrenal-driven Cushing’s syndrome, autonomous cortisol production suppresses ACTH through negative feedback, which may, in turn, inhibit other ACTH-dependent steroid pathways. However, in ACTH-dependent CD, where ACTH excess drives cortisol overproduction, the steroid response may differ fundamentally. Our findings of elevated NS in CD, despite concurrent hypercortisolism, support this mechanistic distinction and provide the first direct evidence of altered NS metabolism in this patient population. Taken together, these observations suggest that the adrenal gland undergoes a functional continuum of dysregulation in CD, from subclinical alterations to overt disease, and that disturbances of the steroidogenic pathway may persist even in states considered biochemically remitted, reflecting the broader and incompletely reversible impact of chronic ACTH-driven hypercortisolism on adrenal steroid metabolism.

PLS-DA identified five NS with VIP > 1: 4-androstenedione (VIP = 2.0), DOC (VIP = 1.8), 7-OH-pregnenolone (VIP = 1.8), corticosterone (VIP = 1.7), and androsterone (VIP = 1.1). The first four were elevated in CD, while androsterone was decreased. Notably, cortisol, E2, and DHT differed between groups (*p* < 0.05) yet showed VIP < 1, whereas DOC exhibited VIP > 1 without univariate significance. This reflects that PLS-DA accounts for intercorrelations: correlated steroids contribute redundant discriminatory information, whereas DOC provides unique classification value.

Beyond these analytical considerations, the overall elevation of multiple steroids alongside cortisol indicates that ACTH-driven adrenal steroidogenesis in CD extends beyond canonical cortisol synthesis to encompass a broader spectrum of steroidogenic pathways.

Steroid profiles showed minimal sex-related differences: testosterone was higher in men within the CD group, as expected, but other sex-dependent steroids, including DHT, E2, and androstenedione, showed no sex differences in either group. Several factors may have contributed to the limited sex differences observed. The mean age of participants (48.2 ± 11.7 years) suggests that many women were perimenopausal or postmenopausal, potentially reducing baseline sex-related variance. Additionally, samples were not timed to the menstrual cycle phase. Although gonadal axis suppression by hypercortisolism may have specifically affected the CD group, the prevalence of hypopituitarism trended higher in patients with CD than in controls (16.2% vs. 0%, *p* = 0.05), which may have further contributed to the limited sex-related differences observed.

Both UFC and ACTH levels showed positive associations with 4-androstenedione, consistent with direct ACTH-driven adrenal synthesis. In contrast, 7-OH-pregnenolone exhibited a negative correlation with ACTH across the CD cohort despite being significantly elevated in CD compared to NFA and exhibiting high discriminatory power (VIP = 1.8). This apparent paradox reflects the distinction between between-group differences and within-group correlations: while ACTH excess in CD overall drives 7-OH-pregnenolone elevation compared to controls, higher ACTH levels within patients with active CD are associated with lower 7-OH-pregnenolone levels, whereas remission patients exhibit paradoxically elevated 7-OH-pregnenolone (121.0 vs. 22.5 ng/mL). This inverse relationship distinguishes 7-OH-pregnenolone from other ACTH-dependent steroids and suggests differential regulation. One possible explanation is substrate rerouting: following ACTH suppression in remission, reduced cortisol synthesis may redirect pregnenolone metabolism toward alternative hydroxylation pathways, including 7α-hydroxylation, resulting in increased 7-OH-pregnenolone accumulation. However, this hypothesis requires confirmation through direct enzymatic and steroidogenic flux analyses in future studies.

7-OH-pregnenolone is a hydroxylated metabolite of pregnenolone synthesized via the enzyme oxysterol 7α-hydroxylase (CYP7B1), expressed in both the adrenal gland and the brain (25). Originally identified as a locomotor-activating NS in lower vertebrates, subsequent studies demonstrated its presence in the mammalian brain, where it modulates dopaminergic and GABAergic neurotransmission and has been implicated in the regulation of locomotor activity and stress responsiveness (25, 26). Although experimental evidence in animal models demonstrates that acute stress increases 7-OH-pregnenolone synthesis through corticosterone-mediated CYP7B1 upregulation (25, 26), its stress-induced regulation in humans remains to be established. Nevertheless, CD represents a state of chronic ACTH-driven hypercortisolism superimposed on recurrent acute stress episodes, which continuously activates the steroidogenic machinery. It is therefore plausible that the positive correlation between 7-OH-pregnenolone and stress scores observed in our cohort reflects a similar corticosterone/cortisol-mediated CYP7B1 activation in the context of chronic ACTH excess. This convergence suggests a potentially distinct role in stress response mechanisms that warrants further investigation in human studies.

Among the discriminatory steroids analyzed, 7-OH-pregnenolone occupied a distinct position: although it emerged as a robust disease discriminator consistent with ACTH-driven adrenal activation, its clinical behavior was distinctly non-canonical. Specifically, its inverse correlation with ACTH levels, paradoxical elevation in remission, and significant association with stress symptoms differentiate it from classical ACTH-dependent steroids. These features confer properties more closely aligned with neuroactive regulation, suggesting that 7-OH-pregnenolone bridges, rather than strictly conforms to, the steroid/NS distinction proposed in this study.

Beyond these correlational patterns, perhaps most striking was the persistence of steroid alterations following disease remission. While cortisol and ACTH normalized as expected, discriminatory steroids remained altered despite biochemical cure. Chronic ACTH excess induces adrenocortical hyperplasia and upregulates steroidogenic enzymes; even after normalization of ACTH, the reversal of adrenal structural and enzymatic remodeling may require a prolonged period (27). The persistence of steroid alterations following biochemical remission likely reflects hypercortisolism-induced epigenetic reprogramming of steroidogenic pathways. Chronic glucocorticoid excess induces lasting epigenetic modifications, including DNA methylation changes and histone modifications that may sustain altered steroid synthesis independently of current ACTH level. Supporting this, genome-wide analyses have demonstrated persistently reduced DNA methylation in patients with CS in remission, with affected genes enriched in hormone receptor pathways (28). These epigenetic fingerprints may explain why discriminatory steroids, including 4-androstenedione and corticosterone, remained elevated in our patients with remitted disease despite normalization of cortisol.

These epigenetic alterations may not only explain persistent steroid dysregulation but also point toward novel therapeutic targets. The reversibility of epigenetic modifications offers a potential therapeutic avenue. Epigenetic drugs termed “epidrugs”, targeting enzymes such as histone deacetylases, have shown promise in experimental models of CS, where histone deacetylase inhibitors attenuated hypercortisolism-induced metabolic and cardiovascular comorbidities (28). Whether such approaches could also reverse NS dysregulation and associated psychiatric morbidity in CD remains to be investigated but represents a compelling direction for future research.

Consistent with previous reports, our patients with CD exhibited significantly higher depression and stress scores compared to NFA controls (29, 30). In contrast to prior studies reporting anxiety prevalence up to 66% in CD (17, 31), we found no significant difference in anxiety scores between groups. This may reflect elevated baseline anxiety in patients with pituitary adenomas regardless of hormonal status, a smaller sample size limiting statistical power, or potential cultural and psychometric factors influencing anxiety reporting in our cohort. We observed no significant correlation between mood symptoms and disease-related characteristics, including cortisol, ACTH, UFC levels, disease duration, remission status, or treatment modality. This finding contrasts with some earlier studies reporting positive correlations between cortisol levels and symptom severity (17, 32, 33), yet aligns with the well-documented persistence of psychiatric symptoms despite biochemical remission observed in 24%–54% of patients (2, 30, 34). Several explanations may account for these observations. First, chronic hypercortisolism induces lasting structural and functional alterations in the brain, particularly in hippocampal and cortical regions involved in mood regulation (32, 33, 35), which may only partially recover after treatment and operate independently of current hormone levels. Second, psychiatric symptoms can precede physical manifestations in up to 12%–27% of cases (36, 37), indicating mood dysregulation independent of peak cortisol exposure. Additionally, hypercortisolism-induced hypomethylation of FKBP5, a key regulator of glucocorticoid receptor sensitivity, has been demonstrated to persist in patients with CS following remission (28, 38). Sustained FKBP5 dysregulation may alter glucocorticoid receptor activity in limbic brain regions, potentially contributing to the dissociation between circulating cortisol levels and psychological symptom burden observed in our cohort.

We identified several significant associations between NS and psychological symptoms that were specific to CD and absent in the NFA group. Among the discriminatory NS, only 7-OH-pregnenolone, which exhibited the unique pattern of increasing in remission and inverse ACTH regulation, correlated with stress symptoms. Notably, the other steroids that best discriminated CD from controls (E2, 4-androstenedione, corticosterone, and androsterone) showed no correlation with mood symptoms, whereas DHEA and allopregnanolone, which did not distinguish groups, exhibited significant associations with mood symptoms. This dissociation suggests that disease markers and psychological symptom markers may operate through distinct pathways. The discriminatory steroids likely reflect ACTH-driven steroidogenic activation that characterizes CD as a disease entity. In contrast, DHEA and allopregnanolone may modulate individual vulnerability or resilience to psychological symptoms within the CD population, potentially through their established neuroactive effects on GABA-A receptors and other neurotransmitter systems. However, this mechanistic hypothesis requires direct experimental validation. This distinction has important implications: targeting cortisol excess alone may achieve biochemical remission without fully addressing the NS-mediated mechanisms underlying persistent psychiatric morbidity.

DHEA showed positive correlations with both depression and stress, while allopregnanolone exhibited negative correlations with both measures. These findings align with the neuroactive properties of these steroids in mood regulation. DHEA, despite its recognized neuroprotective and antidepressant properties in some contexts (39, 40), has shown paradoxical associations with disturbed mood in chronic stress states, potentially reflecting compensatory upregulation or altered metabolism under sustained HPA axis activation (41–43). Conversely, allopregnanolone acts as a positive allosteric modulator of GABA-A receptors, enhancing inhibitory neurotransmission that is critical for stress response regulation (44). Reduced allopregnanolone levels have been consistently associated with major depression (7), and the synthetic analog brexanolone is FDA- and EMA-approved for postpartum depression (12, 14). The negative correlation we observed between allopregnanolone and mood symptoms supports this neuroprotective role and suggests that preserved or enhanced GABAergic NS tone may buffer against psychological morbidity in CD. Critically, these NS–mood associations were absent in the NFA control group, suggesting that the neuroactive effects of these adrenal-derived steroids are specifically altered in the context of ACTH-driven hypercortisolism.

This study has several notable strengths. This is the first comprehensive evaluation of circulating NS profiles in patients with CD, integrating biochemical, psychometric, and clinical data to explore their interrelationships. Importantly, NS levels were quantified by mass spectrometry, a reliable, highly sensitive method that yields accurate measurements with minimal cross-reactivity. The use of a well-matched control group of patients with NFA allowed us to isolate the effects of ACTH-driven hypercortisolism from those related to pituitary pathology and neurosurgical evaluation *per se*. The application of multivariate analysis (PLS-DA) enabled the identification of discriminatory NS, while correlation analyses revealed dissociations between disease biomarkers and symptom-associated NS.

Several limitations warrant consideration. First, the cross-sectional design precludes causal inference and does not capture dynamic changes in NS levels or psychological outcomes over time. Second, the relatively small sample size from a single tertiary center may limit statistical power and generalizability; however, given the low incidence of CD (approximately 1–2 per million per year), our cohort of 37 patients represents a substantial clinical dataset for this rare endocrine disorder. Third, NS samples were not timed according to menstrual cycle phase, and we did not measure aromatase activity or body composition, which limited our ability to explore sex-specific effects or peripheral aromatization mechanisms. Fourth, while DASS-21 is a well-validated instrument, self-reported measures are inherently subject to response bias.

In conclusion, this study demonstrates that CD is characterized by a distinct and persistent steroid signature that extends beyond biochemical remission, reframing CD not solely as a disorder of cortisol excess but as a condition that fundamentally reshapes the epigenetic landscape of steroidogenic pathways. Chronic ACTH excess induces structural and functional adrenal remodeling, and hypercortisolism-driven epigenetic modifications may perpetuate altered steroid synthesis independently of current hormone levels. Notably, while traditional biomarkers (cortisol, ACTH, and UFC) showed no correlation with psychological symptoms, DHEA and allopregnanolone correlated with mood exclusively in patients with CD, suggesting that the relationship between NS and mood is specifically altered in the context of chronic ACTH-driven hypercortisolism. The dissociation between ACTH-driven steroid alterations and mood-associated NS suggests that cortisol normalization alone may be insufficient to restore the NS milieu required for psychiatric recovery. Given that epigenetic modifications are pharmacologically reversible, epigenetic-targeted approaches and NS-based interventions warrant investigation as adjunctive therapies for persistent psychological morbidity in CD.

## Data Availability

The raw data supporting the conclusions of this article are not readily available due to institutional and patient privacy restrictions. However, the datasets will be made available by the corresponding author upon reasonable academic request.
